# ‘Collecting human samples was very hard owing to the fear of sorcery’

**DOI:** 10.1098/rstb.2008.4034

**Published:** 2008-11-27

**Authors:** Koiye Tasa

**Affiliations:** Asavinti Hamlet, Emo Village, c/o Papua New Guinea Institute of Medical ResearchPO Box 60, Goroka, EHP 441, Papua New Guinea

I was less than 10 years old when Dr Carleton Gajdusek came into the Purosa Valley to carry out his research on kuru disease. Michael Alpers came after him and I worked alongside them to patrol out into the villages that were affected by kuru. My job was mainly to accompany Carleton and assist in collecting kuru patients' samples and diagnosing their disease. I also worked as a kuru surveillance officer and went on my own into the villages to locate kuru patients. Sometimes, I was given the task by Carleton of transporting brain tissue and other selected internal organs to Kainantu station by tractor or Landrover.

The people's belief system was that kuru was caused by sorcery as a means of payback by other clan members within the village or a nearby village. Collecting human samples was very hard owing to the fear of sorcery: the people feared that we might misplace some of the samples and sorcerers might pick them up. Nevertheless, those of us who worked with western medical scientists were free to move around in the villages to assist kuru patients and their families.

The others that I worked with in the field include John Mathews, John Colman and Jack Baker. It is great to meet again some of those colleagues I once worked with in the South Fore area of Okapa District.

